# Toxicity of *Bacillus thuringiensis*-Derived Pesticidal Proteins Cry1Ab and Cry1Ba against Asian Citrus Psyllid, *Diaphorina citri* (Hemiptera)

**DOI:** 10.3390/toxins11030173

**Published:** 2019-03-22

**Authors:** Maria Teresa Fernandez-Luna, Pavan Kumar, David G. Hall, Ashaki D. Mitchell, Michael B. Blackburn, Bryony C. Bonning

**Affiliations:** 1 Department of Entomology, Iowa State University, Ames, IA 50011, USA; maria_fernandez-luna@baylor.edu; 2 Department of Entomology and Nematology, University of Florida, Gainesville, FL 32611, USA; pavankumarf@ufl.edu; 3 U.S. Horticultural Research Laboratory, USDA ARS, Fort Pierce, FL 34945, USA; David.Hall@ARS.USDA.GOV; 4 Invasive Insect Biocontrol and Behavior Laboratory, USDA ARS, Beltsville, MD 20705, USA; Teddi.Mitchell@ARS.USDA.GOV (A.D.M.); Mike.Blackburn@ARS.USDA.GOV (M.B.B.)

**Keywords:** Asian citrus psyllid, *Bacillus thuringiensis*, pesticidal protein, toxin, gut epithelium

## Abstract

The Asian citrus psyllid (ACP), *Diaphorina citri* Kuwayama (Hemiptera), is an important pest of citriculture. The ACP vectors a bacterium that causes huanglongbing (HLB), a devastating and incurable disease of citrus. The bacterium *Bacillus thuringiensis* (Bt) produces multiple toxins with activity against a diverse range of insects. In efforts to provide additional control methods for the ACP vector of HLB, we identified pesticidal proteins derived from Bt for toxicity against ACP. The trypsin proteolytic profiles of strain-derived toxins were characterized. Strain IBL-00200, one of six strains with toxins shown to have basal activity against ACP was selected for liquid chromatography-mass spectrometry (LC-MS/MS) identification of the individual Cry toxins expressed. Toxicity assays with individual toxins derived from IBL-00200 were then performed. The activated form of the Cry toxins Cry1Ab and Cry1Ba were toxic to ACP with LC_50_ values of approximately 120 µg/mL. Disruption of the midgut epithelium was associated with the toxicity of both the IBL-00200-derived toxin mixture, and with Cry1Ba. With further optimization of the efficacy of Cry1Ab and Cry1Ba, these toxins may have practical utility against ACP. Bt toxins with activity against ACP may provide an additional tool for management of ACP and the associated HLB disease, thereby providing a more sustainable and environmentally benign approach than repeated application of broad-spectrum insecticides.

## 1. Introduction

The Asian citrus psyllid (ACP), *Diaphorina citri* Kuwayama (Hemiptera: Psyllidae), is one of the most important pests of citrus worldwide because it transmits a pathogenic bacterium responsible for citrus huanglongbing (HLB) disease (also known as citrus greening). Most types of citrus, especially sweet orange and grapefruit, are susceptible to the disease. Trees infected by the bacterium develop HLB symptoms within 2 to 3 years, and decline in health and productivity to the point of being economically unviable [1,2]. Severe economic losses are attributed to HLB in citriculture, a disease that is difficult and costly to manage [3,4,5].

Intensive insecticide application programs against the ACP are currently advocated for preventing HLB in citrus [1,6]. However, long-term use of insecticides against ACP is not considered sustainable for both economic and environmental reasons. Furthermore, development of insecticide resistance in ACP has already been reported in Florida [7,8].

One possible alternative to chemical insecticides for ACP management is the use of pesticidal proteins derived from *Bacillus thuringiensis* (Bt), a gram-positive, spore-forming bacterium. Bt *δ*-endotoxins, which include the crystal (Cry) proteins, damage the midgut post ingestion and have been used successfully for management of other insect pests [9,10]. The Cry proteins are pore-forming toxins, although the mechanism of action is incompletely understood [11,12]. While individual Cry toxins are generally toxic to a particular order of insects, collectively they exhibit activity across orders, particularly the Lepidoptera, Diptera and Coleoptera. A major limitation for use of Bt toxins against ACP is the lack of information on the efficacy of Bt toxins against ACP. Some Bt toxins have activity against sap sucking insects with, for example, low-level toxicity against aphids (Hemiptera: Aphididae) [13], and high toxicity following optimization against plant bugs, *Lygus hesperus* (Hemiptera: Miridae) [14,15]. An ACP-active toxin could be delivered to the ACP feeding site (primarily the phloem) via use of transgenic citrus, or use of a non-pathogenic phloem-inhabiting bacterium or virus such as the *Citrus tristeza virus* vector [16].

The overall goal of this study was to identify a Bt crystal toxin with basal toxicity to ACP. Having demonstrated that the addition of gut-binding peptides to toxins with basal activity can enhance toxin efficacy against hemipteran pests [17], these toxins will be optimized for use in the field. Toxins derived from Bt could be used for effective management of the psyllid and associated HLB disease to the benefit of both the citrus industry and the environment as a more sustainable management approach than the excessive use of non-specific chemical insecticides.

## 2. Results

### 2.1. Bt Strain-Derived Toxins with Activity against Asian Citrus Psyllid (ACP)

To identify Bt strain-derived toxins that have toxicity against ACP, toxin mixtures derived from each of 18 Bt strains and two recombinant Bt Cry toxins were screened in ACP bioassays using toxin preparations that had been solubilized and trypsin activated to expose ACP to activated toxins (Table 1). Of the 18 Bt isolates tested, six strains expressed toxins that were toxic to ACP, the two recombinant Cry toxins –Cry4A and Cry11A were not toxic, and 12 strains lacked toxicity (Table 1). For the six isolates with significant ACP mortality compared to control treatments, a toxin dose of 500 μg/mL resulted in mortality at day 7 ranging from 50% to 100%. Mortality induced by toxins derived from each Bt strain (normalized with the control mortality) at day 7 was: IBL-68, 70%; IBL-365, 60%; IBL-681, 50%; IBL-200, 45%; IBL-48, 40%; IBL-829, 30%. The estimated probability of mortality at day 7 showed significantly increased probability for these six treatments compared to the control by logistic regression analysis.

### 2.2. Toxicity Correlated with Reduced Feeding

The intoxication effects of Cry toxins include disruption of the midgut epithelium resulting in gut paralysis, cessation of feeding, starvation and eventual death [18]. Excretions produced as psyllids feed [19] were monitored as an indicator of feeding cessation. Based on this assay, adult ACP stopped feeding on some Bt strains (Supplementary Figure S1). Specifically, ACP fed on toxins derived from strains IBL-00068, -00200, and -00829 at a dose of 500 μg/mL produced significantly fewer excretions compared to the buffer control diet after 7 days of exposure (Dunn’s test; *p* < 0.05. All insects in the IBL-00365 bioassay were dead by day 7 such that data for this strain could not be included in the analysis). This result suggests that mortality recorded in these assays resulted from cessation of feeding induced by the action of Cry toxins.

### 2.3. Toxin Proteolytic Profiles

**Figure 1:**
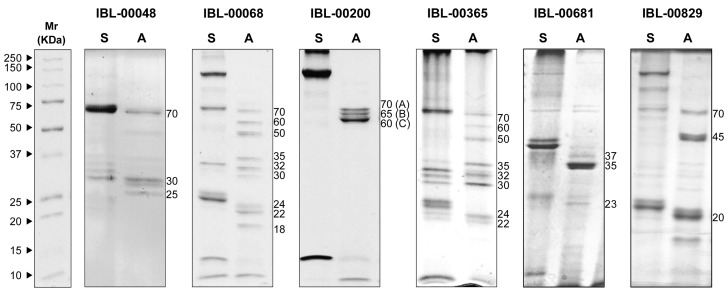
Profiles of trypsin-activated toxins derived from *Bt* isolates with toxicity to ACP: S: Soluble protein. A: Activated protein treated with 10% Trypsin for 1 h at 37 °C. Proteins were separated by sodium dodecyl sulfate polyacrylamide gel electrophoresis (SDS-PAGE) (12% gel) and the gel stained with Coomassie Blue R. Images of gels from which lanes were taken are presented in Figure S2.

Solubilization and activation of Bt strain-derived toxins generated different proteolytic profiles, with a total of 10 distinct proteolytic profiles (data not shown) identified from the toxin strains examined. Profiles of trypsin-activated toxins are shown for strains with toxicity to ACP (Figure 1; Figure S2). The pro-toxin protein profile (i.e., soluble protein) of the majority of the Bt isolates produced bands of approximately 25, 40, 75 and/or 120 KDa. The molecular mass of the trypsin-activated toxins ranged from 20 KDa to 70 KDa, suggesting a diverse composition of Cry and possibly also Cyt toxins.

### 2.4. *Bt israelensis* Strains

Toxins derived from strains IBL-00048, IBL-00068 and IBL-00365 showed the highest levels of toxicity against ACP. Sequencing and annotation of the genomes of these three strains (M. Blackburn, unpublished data) suggests toxin similarity to those expressed by *Bacillus thuringiensis* subsp. *israelensis* (Bti). The profiles of activated toxins from strains IBL-00068 and IBL-00365 were similar, suggesting that these two strains express a similar toxin combination. BLASTn analysis indicated that all three strains express Cry4, Cry10 and Cry11. As neither Cry11A nor Cry4A were toxic against ACP (Table 1), there may be a synergistic effect between the Cry and cytolytic (Cyt) toxins expressed by Bti, as previously observed by Fernandez-Luna et al. [20]. While Cyt toxins may have toxicity against *D. citri*, they are not of interest for *D. citri* management due to their lack of binding specificity.

### 2.5. Identification of Toxins Expressed by IBL-00200

Strain IBL-00200 was selected for analysis of the toxicity of individual toxins produced by that strain. The proteolytic profile after trypsin treatment of toxins derived from IBL-00200 shows three bands of different molecular mass (A, B, C; Figure 1). These bands were cut from the gel and submitted for peptide sequencing and toxin identification (Supplementary Figure S3). Peptide sequences obtained from the bands were found to be consistent with the translated products of three genes found on a 55 kb fragment of the partially assembled IBL-00200 genome (GenBank: ACNK01000108.1). Peptides obtained from bands A, B, and C were consistent with GenBank proteins EEM92927.1, EEM92947.1, and EEM92934.1 respectively, all of which are encoded on the 55 kb fragment. EEM92927.1 was annotated as a cry1Bc, while both EEM92947.1 and EEM92934.1 were annotated as cry1Ae. However, maximum likelihood analysis (Supplementary Figure S4) of the predicted protein sequences with those of holotype cry1 toxins [21], reveal that EEM92927.1 is most closely related to cry1Bb, while EEM92947.1 is most closely related to cry1Ja, and EEM92934.1 to cry1Ab (Table 2; Table 3). Thus, the tryptic fragments A, B, and C observed in Figure 1 are due to proteins highly similar to Cry1Bb, Cry1Ja, and Cry1Ab, respectively, and the 120 kDa band in the soluble protein fraction (Figure 1) is comprised of multiple toxins of similar size. It is notable that although the IBL-00200 genome appears to include a number of other cry genes, these three were the only ones detected in the parasporal crystal. Supplementary Figure S3 shows some of the peptide sequences obtained from bands A, B and C along with perfect matches to IBL-00200 genes. As there is a high degree of sequence conservation between Cry toxins, a few peptides produced matches to multiple predicted proteins, but in all of these cases one of the matches was also one of the three proteins described above.

### 2.6. Toxicity of Individual Toxins to ACP

**Figure 2:**
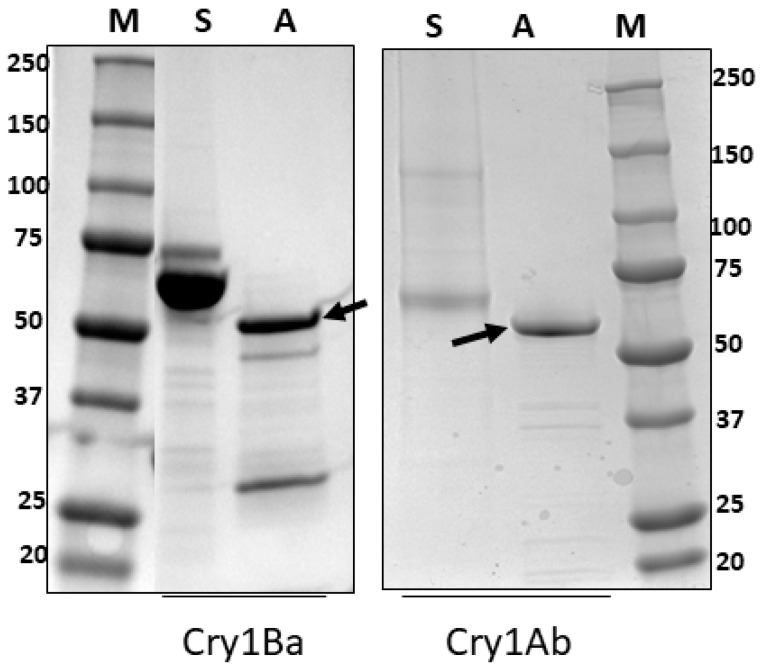
Activation of purified Cry1Ba and Cry1Ab. SDS-PAGE (4%–20% gradient gel) separation of solubilized (S) and activated (A) Cry1Ba and Cry1Ab. Molecular mass markers are shown (M). Proteins of the expected size for activated Cry1Ab (62 kDa) and Cry1Ba (55 kDa) are indicated by arrows.

To understand the contribution of individual toxins derived from IBL-00200 to the ACP toxicity induced by this strain, we tested activated Cry1Ab and Cry1Ba (Figure 2, Figure S5) for toxicity against ACP. Constructs for expression of these toxins were kindly provided by Drs. M. Adang, University of Georgia and Dr. R.de Maagd, Wageningen University. However, as Cry1Ba has only 83% amino acid identity to Cry1Bb, it cannot be assumed that Cry1Ba and Cry1Bb have the same impact on ACP. The LC_50_ values for Cry1Ab and Cry1Ba against adult ACP after 5 days of feeding were comparable at 118 and 125 µg/mL respectively (Table 4; *t*-test, *p* = 0.77). A significant reduction in excretions was noted for ACP fed on Cry1Ab and Cry1Ba after 11 days of exposure when compared to the Buffer control (Dunn’s test; *p* < 0.05; Supplementary Figure S1). This result, with significant differences recorded at 11 days rather than at 7 days as for strain-derived toxin mixtures, suggests that the combined action of toxins derived from IBL-00200 resulted in feeding cessation more rapidly than the individual components tested.

### 2.7. Toxicity Correlates with Damage to the ACP Gut Epithelium

**Figure 3:**
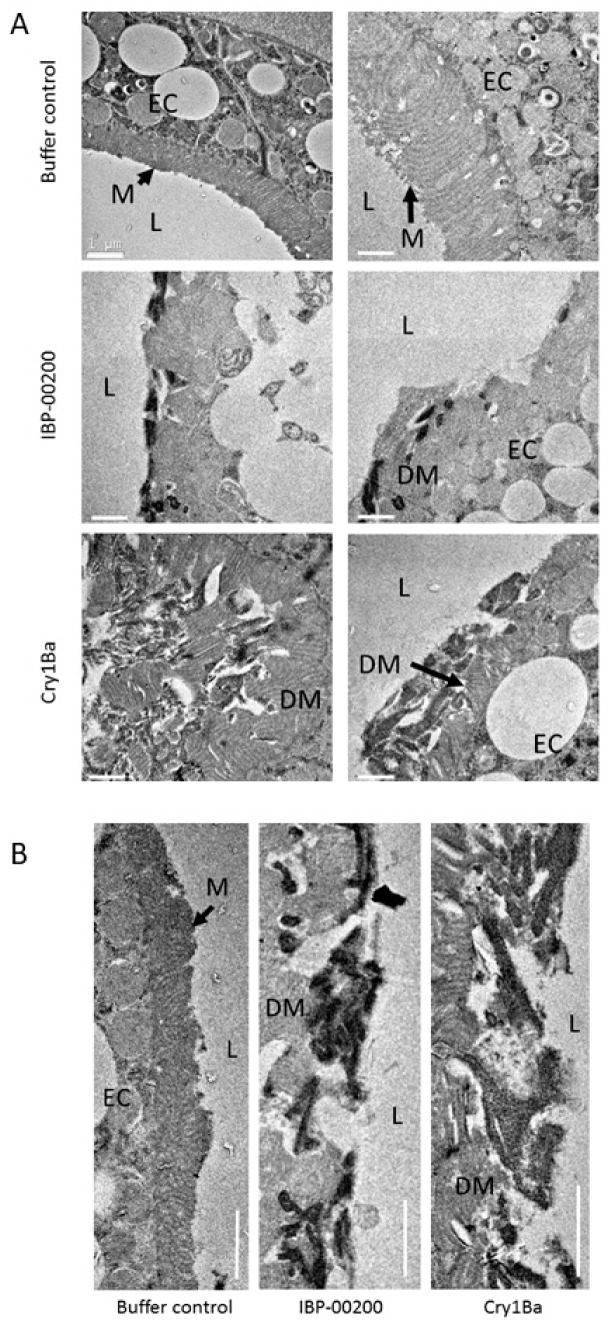
Toxins cause extensive damage to ACP midgut epithelium. Transmission electron micrographs of midguts of ACP showing the impact of toxins derived from strain IBP-00200, or of Cry1Ba alone on the midgut epithelium relative to the control (buffer). (**A**) Impact of treatments on microvilli (M) projecting into the gut lumen (L) and on the underlying epithelial cells (EC), with two representative images shown for each treatment. (**B**) Detail of microvilli, with loss of integrity in toxin treatments. DM, disrupted microvilli. Bars represent 1 µm.

Ingestion of activated toxins derived from IBL-00200, and of activated Cry1Ba resulted in widespread damage to the ACP midgut epithelium (Figure 3). While gut microvilli in control insects appeared densely packed and regular in organization, microvilli had disintegrated in ACP fed on IBP-0200-derived toxins. The microvilli of insects fed Cry1Ba were disorganized, and detached from the cell surface and cell debris was evident in the gut lumen.

## 3. Discussion

The lethal action of Bt toxins is dependent on the acid or alkaline pH of the gut of the target insect, the action of proteolytic enzymes, and the presence of appropriate midgut microvillar receptors. In susceptible species, toxins are processed by the pH and proteases in the midgut lumen from insoluble protoxins within crystalline inclusions to the soluble, toxic active form that is resistant to further proteolysis [11,12,18]. The lack of susceptibility of some insects to Bt toxins can be attributed to differences in physiology compared to susceptible species [13].

Limited research has been done on identification of Bt toxins with activity against Hemiptera [13]. A Cry1 toxin mixture, Cry2Aa, Cry3Aa and Cry11Aa have been shown to be toxic against the potato aphid (*Macrosiphum euphorbiae*) [22]. In that report the authors demonstrated that a mixture of Cry1Aa, Cry1Ab, Cry1Ca and Cry1Fa resulted in 100% mortality after 96 h [22], compared to only 12% mortality with the single Cry1Ac after 120 h. In the present study we demonstrated that Cry1Ab is toxic to ACP. Cry1Ab also showed moderate toxicity against the pea aphid (*Acyrthosiphon pisum*) at a dose of 500 µg/mL of solubilized protein, corroborating the basal levels of toxicity against pea aphids as well as against ACP [23]. Solubilized and trypsin-activated forms of four Cry δ-endotoxins (Cry1Ab, Cry3A, Cry4Aa and Cry11Aa) were toxic to the pea aphid at 500 µg/mL of solubilized protein [23]. Cry4Aa and Cry11Aa each resulted in 100% pea aphid mortality after 3 days at 500 μg/mL, and Cry3 induced 60% mortality at day 6. Finally, although several Cry1 toxins have low toxicity against hemipteran insects, the toxicity of Cry1Ba against ACP is the first report of toxicity for this toxin against a hemipteran.

In the study of Walters and English [22], Cry2 resulted in 47% potato aphid mortality (24 h), Cry3 in 52% and Cry11 in 64% mortality after 72 h. An important difference in the present study compared to Walters and English [22], is that the ACP mortality was due to toxins activated in vitro, suggesting that Cry2 and Cry3 may be more toxic toward hemipterans if subjected to trypsin activation. Furthermore, while Cry11A showed toxicity against the potato aphid [22], Cry11A was not toxic to ACP. The toxicity of a few β pore-forming toxins against hemipteran pests has also been demonstrated [24,25], with toxicity levels of Cry51Aa2 optimized for efficacy against *Lygus* spp. for commercial use in transgenic cotton [25]. Although bioassay conditions differed, it is of note that the toxicity of Cry51Aa2 against *Lygus hesperus* at 73 ppm (95% confidence interval of 50–116) [15] prior to optimization for use in cotton was comparable to the toxicity of Cry1Ab and Cry1Ba against ACP shown here.

Annotation of the genome sequence of Bt strain IBL-00200 was conducted by use of a computer generated database extracted from GenBank rather than with reference to the Bt toxin nomenclature database. We determined which toxin genes produced the observed peptides, and compared toxins to the original (holotype) Cry toxins from [21,26] by using the maximum likelihood method. Analysis of the maximum likelihood tree, allowed for identification of Cry proteins nearest to the IBL-00200 proteins that were identified by MS/MS peptide sequencing. Our work has demonstrated that these toxins are expressed by this strain and the Cry designations are accurate.

Different Cry toxins may interact in the insect gut resulting in synergism, additive and/or antagonist effects on mortality [20,27]. In the present study, Cry1Ab was the most abundant toxin produced by IBL-00200 following trypsin activation (Figure 1). While the bioassays reported here were conducted with adult insects, it is expected that nymphs will be considerably more susceptible to these toxins based on greater susceptibility to toxin impacts and to the large volumes of sap ingested during the nymphal stages. In addition, toxin modification for further enhancement of toxicity against ACP can be employed to attain commercially relevant toxicity levels [17,25].

Effective strategies are urgently needed for management of ACP-vectored HLB. As transgenic citrus trees are slow and difficult to produce, an alternative approach would be required for delivery of an ACP-active toxin to the plant phloem for ingestion by ACP. The plant virus, Citrus tristeza virus (CTV) has been developed as an effective vector for delivery of bioactives to ACP and appears to be stable in citrus for long periods of time (i.e., decades) [28]. CTV could provide an appropriate delivery system for ACP-active toxins to the phloem, and such toxins could potentially be expressed alongside other bioactives, such as gene silencing RNAs in a single CTV vector.

## 4. Materials and Methods

### 4.1. *Bacillus thuringiensis* (Bt) Strains and Toxins

*Bacillus thuringiensis* strains used in this study were selected from the collection of the Invasive Insect Biocontrol and Behavior Laboratory (USDA-ARS, Beltsville, MD, USA). Bt spores stored on paper disks were germinated by placing the disks on L-agar at 30 °C. At 48 h, vegetative cells from the edge of the disks were sub-cultured twice consecutively for 72 h at 30 °C on T3 agar as described by Travers et al. [29]. At the end of each subculture, colonies were checked microscopically to verify that the cultures had sporulated and that parasporal crystals had been produced. After the second subculture, five plates of T3 agar were inoculated and allowed to grow at 30 °C for at least 96 h. After a final microscopic examination to verify sporulation and crystal production, 10 mL of sterile water was pipetted onto each of the five plates, and a sterile metal spreader was used to gently remove the colonies from the plate. The resulting spore and crystal suspension was removed from the plate using a 5 mL pipette and placed into sterile, disposable 50 mL conical centrifuge tubes. The Cry1Ab from *Bacillus thuringiensis kurstaki* HD1 was produced in *E. coli* on LB medium with ampicillin (40 μg/mL) at 37 °C with shaking for 24 h, according to Geiser et al. [30]. Cry1Ba was produced in a *Bacillus thuringiensis* strain in a Cry minus background [31], with NBY Medium [32] at 30 °C with shaking at 180 rpm for 72 h or until sporulation and crystal formation was evident.

### 4.2. Solubilization, Trypsin Activation and Toxin Profiles of Bt Strains

Harvested spores and crystals were washed three times with 300 mM NaCl, 10 mM ethylenediaminetetraacetic acid (EDTA), pH 8.0, supplemented with PMSF 0.1 mM. Enriched Cry toxin pellets from each Bt strain tested were solubilized and activated with trypsin according to Fernandez-Luna et al. [20] with minor modifications. For the solubilization, the crystals and spores were pelleted at 12,000× *g* for 20 min at 4 °C. The pellet was resuspended and sonicated in 10 mL buffer (50 mM Tris-HCl pH 8 supplemented with Lysozyme 200 μg/mL). The pellet was sonicated three times for intervals of 10 s and 1 min in ice. The crystals and spores were spun down again at 12,000× *g* for 20 min at 4 °C. The crystals were then solubilized by resuspending the pellet in 12 mL of 50 mM sodium carbonate (pH 10.5) with 5 mM dithiothreitol (DTT) and incubated for 3 h at 37 °C with 120 rpm orbital shaking. After solubilization, samples were centrifuged at 12,000× *g* for 10 min at 4 °C to pellet the spores and non-soluble material. The solubilized sample was dialyzed against 50 mM Tris-HCl pH 8.8 with three buffer exchanges (1 L each), and protein quantified [33] using bovine serum albumin as standard. Due to the lack of information about ACP digestive gut proteases, which are essential for activation of Bt toxins in the insect gut, in vitro activated Cry toxins were used in ACP feeding assays. Trypsin from bovine pancreas (tosyl phenylalanyl chloromethyl ketone –TPCK treated; Sigma-Aldrich, St. Louis, MO, USA) was used at a 10:1 ratio (*w*/*w*) of toxin:trypsin for activation. The reaction was conducted for 1–2 h at 37 °C with 180 rpm orbital shaking. Trypsin was removed by trypsin affinity matrix (Benzamidine Sepharose 6B, GE Healthcare, Marlborough, MA, USA) before use in ACP feeding assays following the protocol of the manufacturer. The toxin profile was examined by sodium dodecyl sulfate polyacrylamide gel electrophoresis (SDS-PAGE) with 12% acrylamide gels. The protein concentrations of toxin samples were quantified and samples stored at −80 °C until use.

### 4.3. Screening of Bt Accessions for Toxicity to Adult ACP

Relative toxicity of toxin mixtures derived from individual strains was investigated by comparing mortality rates of ACP feeding on a fixed concentration of 500 μg/mL Bt in a liquid diet to mortality of ACP feeding control diet. The ACP were obtained from a colony previously described [34,35] with bioassays being conducted at USDA ARS, Fort Pierce, FL. Briefly, the psyllids were continuously reared in a greenhouse on *Citrus macrophylla* Wester, a genotype favored by ACP for colonization [36]. The colony was maintained using procedures similar to those described by Skelley and Hoy [37] and tested quarterly to ensure that the colony remained HLB-free.

The initial base diet for adult psyllids [34] consisted of sucrose (30%), yellow food coloring (0.4%), and green food coloring (0.1%) in tap water, with diet autoclaved after mixing. Addition of trypsin-activated Bt toxins to this diet at a concentration of 500 μg/mL resulted in precipitation. Subsequently, food coloring was excluded from the psyllid diet to avoid precipitation and a sachet of green diet placed behind the sachet of clear diet with Bt to attract ACP to the diet.

Five adult psyllids (≤7 days-old) per plastic vial were subdued by cooling at 5 °C. Vials were removed from the refrigerator one at a time, and psyllids placed into the feeding chamber dish in a clean air hood. A 4.8 × 2.5 cm piece of Parafilm^®^ membrane was stretched across the dish above the psyllids. A slight indentation of the membrane was created with a sterile gloved finger in the center of the membrane, and 0.5 mL of the liquid diet was pipetted into this depression. A second membrane was then stretched across the first, sandwiching the diet. The sachet of green diet was then placed on top of the sachet of clear diet. Feeding chambers were then placed in a growth chamber at 25 °C, 14:10 h light: dark and 75% relative humidity.

Samples of activated toxin mixtures from individual Bt strains were tested for toxicity, with five adult psyllids per feeding chamber and four feeding chambers (technical replicates) per toxin. Control dishes included sugar diet only (Diet Control) and sugar diet with the same volume of 50 mM Tris-HCl pH 8 added as for the toxin samples (Buffer Control), to control for possible buffer effects on psyllid survival. Four technical replicates, each with 5 psyllids per dish, were conducted for each bioassay (*n* = 20 psyllids total per treatment). Between one and four biological replicates were conducted for each bioassay. Mortality was scored daily.

Statistical significance of mortality resulting from ingestion of strain-derived toxin mixtures was assessed using one-way analysis of variance (ANOVA) and Student’s *t* tests to evaluate differences between groups, with the use of GraphPad Prism version 5.00 for MacOSX (GraphPad Software, San Diego, CA, USA). A *p*-value of less than 0.05 was considered statistically significant unless otherwise indicated. If an overall significance was detected, Tukey’s multiple range tests were performed. All data were analyzed prior to statistical analysis to meet the homoscedasticity and normality assumptions of parametric tests.

On the third, seventh and eleventh days of each bioassay, the relative quantity of ACP excretions present in each feeding chamber was rated (0: none; 1: <5 droppings; 2: ≥5 droppings). This analysis was done for one to three of the biological replicates with a minimum of 20 ACP scored per treatment. For calculation of significant differences in excretions between test and buffer control treatments at different times post-exposure, non-parametric tests were employed. Groups were analyzed using the Kruskal–Wallis test and major differences observed were examined further using Dunn’s two-tailed nonparametric multiple comparison test (GraphPad Prism version 5.00 for MacOSX). A *p*-value of less than 0.05 was considered statistically significant.

### 4.4. Identification of Toxins Produced by IBL-00200

Bt strain IBL-00200 was among the strains identified as having toxicity to ACP. The solubilized and trypsin-activated toxins were separated by SDS-PAGE (12% gel), protein bands isolated and treated with trypsin for protein identification through liquid chromatography-mass spectrometry (LC-MS/MS). The translated IBL-00200 genome was used as reference for identification of proteins based on trypsin peptide profiles generated by MS/MS, using Thermo Scientific Proteome Discoverer software. Maximum likelihood analysis of protein sequences was performed with MEGA 6 [38] using the default parameters.

### 4.5. Expression and Purification of Cry1Ab and Cry1Ba

For expression of Cry1Ab, the Bt serovar *kurstaki* HD1-9 strain was obtained from the Bacillus Genetic Stock Center, Columbus, OH. Cry1Ab was expressed as reported by Zhang et al. [39]. A single colony of HD1-9 strain was grown in medium containing 2 gm/L peptone 5 gm/L yeast extract, 0.07 M K_2_HPO_4_, 0.02 M KH_2_PO_4_, 6 × 10^−3^ M glucose, 2 × 10^−4^ M MgSO_4_·7H_2_O, 5 × 10^−4^ M CaCl_2_·2H_2_O, 6 × 10^−6^ M MnSO_4_·7H_2_O and 1 × 10^−6^ M FeSO_4_·7H_2_O with erythromycin (20 µg/mL) overnight at 30 °C. After 24 h an equal volume of sterile sodium phosphate solution (0.06 M Na_2_HPO_4_, 0.04 M NaH_2_PO_4_·H_2_O) was added and bacteria grown for another three days when 100% sporulation was achieved. The spores and crystals were harvested, sonicated and washed three times with 1 M NaCl and distilled water by suspending and centrifugation at 10,000× *g* at 4 °C for 10 min. The pellet, containing protoxin in the form of crystals, was solubilized using 20 mM Na_2_CO_3_ buffer, pH 10.0 containing 20 mM EDTA and 10 mM DTT at 37 °C overnight. The clear supernatant was collected after centrifugation at 10,000× *g* for 10 min at RT. The solubilized protein, present in the collected supernatant, was activated by treating with bovine trypsin (Sigma, St. Louis, MO, USA) at a 1:10 ratio of trypsin: protein, after adjusting the pH to 7.5 using 1 N HCl, for 2 h at 37 °C. The total protein in the activated fractions was estimated using Bradford’s Reagent and proteins were separated by SDS-PAGE in 4%–20% gradient gels to confirm the protein size.

For expression of Cry1Ba, *E. coli* XL-1 cells were transformed with the plasmid pMH19 [40]. A single transformed colony was grown on TB-medium containing ampicillin (100 μg/mL) and 2% glucose at 37 °C overnight. This pre-culture was inoculated into fresh medium and further grown at 28 °C for 3 days. The cells were harvested and the pellet suspended in lysis buffer (50 mM Tris-HCl pH8.0, 5 mM EDTA, 100 mM NaCl,). The pellet was further washed three times with buffer (20 mM TrisHCl, pH 7.5, 1 M NaCl, 1% Triton X-100) and the crystals were solubilized in 50 mM Na_2_CO_3_ buffer, 20 mM NaCl, 10 mM DTT pH10. The solubilized Cry1Ba was activated, quantified and analyzed as described above.

### 4.6. Screening of Individual Bt Endotoxins for Toxicity to Adult ACP

Bioassays to assess the toxicity of individual toxins against ACP were performed at the University of Florida as reported earlier with modifications [41,42]. A 30% sucrose solution-based diet in sterile water containing 0.1% green food dye and 0.4% yellow food dye (McCormick & Co., Inc., Hunt Valley, MD, USA) was prepared with 12.5, 25, 50, 100, or 200 µg/mL of toxin or with the respective buffer control. A diet without toxin or buffer was used as an additional negative control. Snap-cap polypropylene tubes (8 mL capacity: Fisher Scientific, Waltham, MA, USA) were used for the bioassay. The lids of these tubes were filled with 0.5 mL of the respective diet and sealed with a 2 cm^2^ piece of Parafilm M^®^ (Bemis^®^, Neenha, WI, USA) by stretching and ensuring that lids were leak-free. The 10-day old adult ACP from a CLas-free colony maintained on Orange jasmine were provided by the Division of Plant Industry, Florida Department of Agriculture and Consumer Services, Gainesville, FL. Five ACP were carefully transferred into each assay vial using a paint brush while exposing them to a white light and the lid closed. Three to five technical replicates for each treatment were conducted and the experiment was repeated for a total of three biological replicates. The tubes were maintained upright in a Percival incubator for five days at 28 °C/ 25 °C with 14: 10 light: dark photoperiod at 70% relative humidity. Mortality was recorded after 5 days and the LC_50_ value for each technical replicate for each toxin calculated by Probit analysis [42]. The mean LC_50_ value from three independent experiments was also calculated.

### 4.7. Transmission Electron Microscopy

Adult ACP were fed by membrane feeding on artificial diet in 30% sucrose-TRIS with trypsin activated toxin extract of IBL-00200 (500 μg/mL), Cry1Ba (500 μg/mL) or Tris buffer alone added. Twenty insects per treatment were fed for 48 h in a growth chamber at 25 °C, 14:10 h light: dark and 75% relative humidity, with two replicate assays. ACP from all replicates were pooled. The distal abdominal segments, head and legs were removed and torsos then immediately fixed (2% paraformaldehyde, 2.5% glutaraldehyde, 0.05 M cacoldylate buffer, pH 7.1) for 48 h at 4 °C. Samples were washed in buffer and then fixed in 1% osmium tetroxide in 0.1 M cacodylate buffer for 1 h. The samples were then en-bloc stained with 2% uranyl acetate for 2 h, dehydrated in a graded ethanol series, cleared with ultra-pure acetone, infiltrated and embedded using a modified EPON epoxy resin (Embed 812; Electron Microscopy Sciences, Ft. Washington, PA, USA). Resin blocks were polymerized for 48 h at 70 °C. Thick and ultrathin sections were made using a Leica UC6 ultramicrotome (North Central Instruments, Minneapolis, MN, USA). Ultrathin sections were collected onto copper grids and images were captured using a JEM 2100 200 kV scanning and transmission electron microscope (Japan Electron Optic Laboratories, LLC, Peabody, MA, USA).

## Figures and Tables

**Table 1:** Bt strains and individual toxins tested for toxicity against Asian citrus psyllid (ACP). Activated and purified toxins from each strain, or individual toxins were tested in bioassays with adult ACP as described. † Single biological replicate.

Toxic	Non-Toxic
IBL-00048 †	IBL-00024
IBL-00068	IBL-00055
IBL-00200	IBL-00071
IBL-00365	IBL-00090
IBL-00681	IBL-00098
IBL-00829	IBL-00192
Cry1Ab	IBL-00217
Cry1Ba	IBL-00438
	IBL-00937
	IBL-01306
	IBL-01313
	IBL-03792
	Cry4A
	Cry11A

**Table 2:** Cry toxins expressed by strain IBL-00200 based on mass spectrometry (MS/MS) data generated. Score A4: sum of the scores of the individual peptides from the Sequest HT search (calculated by: 0.8 + peptide charge × peptide relevance factor). Data were analyzed by Proteome Discoverer software V2.1 for Windows (Thermo Scientific, Waltham, MA, USA). See Supplementary Figure S3 for peptides sequenced.

Band	Accession Number	Identified Cry Toxin	Coverage (%)	Amino Acid Identity (%)	Score A4
A	EEM92927.1	Cry1Bb	21.93	99.9	360.29
B	EEM92947.1	Cry1Ja	32.39	99	674.73
C	EEM92934.1	Cry1Ab	30.59	96	698.91

**Table 3:** Toxins encoded by Bt strain IBL-00200 according to the genome sequence (GenBank Accession CM000758.1) and de novo identification of the expressed toxins under sporulation conditions based on the Bt toxin nomenclature database [21].

Accession Numbers	Previous Designation	Expression in IBL-00200 and Designation Based on Bt Database	Locus_tag
EEM93105.1	cry11Bb	ND	
EEM93049.1	cry2Ad	ND	
EEM93050.1	cry2Ad	ND	
EEM93051.1	cry1Ae	ND	
EEM93055.1	cry2Ad	ND	
EEM93056.1	cry1Ae	ND	
EEM92924.1	cry1Ae	cry1Hb	
EEM92927.1	cry1Bc	cry1Bb	bthur0013_56890
EEM92934.1	cry1Ae	cry1Ab	bthur0013_56960
EEM92941.1	cry1Bc	ND	
EEM92947.1	cry1Ae	cry1Ja	bthur0013_57090
EEM92952.1	cry1Bc	ND	
EEM92953.1	cry1Ae	cry1Da	
EEM92570.1	cry8Ba	ND	

**Table 4:** Cry1Ab and Cry1Ba1 toxicity against adult ACP. The LC_50_ value of Cry1Ab and Cry1Ba from three independent experiments (*n* = 15 to 30 ACP per toxin concentration per experiment) are shown with lower and upper 95% confidence intervals (CI). The mean (+/− SE) LC_50_ values were not significantly different (*p* = 0.77; two tailed *t*-test).

Toxin	LC_50_ (µg/mL or ppm)	95% Fiducial CI		Mean LC_50_	Standard Error
		Lower	Upper		
Cry1Ab	123	56	272	118	17.1
	149	89	250		
	102	51	206		
Cry1Ba	95	29	310	125	13.6
	108	58	202		
	152	77	300		

## Supplementary Materials

The following supplementary materials are available online at https://www.mdpi.com/2072-6651/11/3/173/s1, Figure S1: Toxicity correlated with reduced ACP excretions, Figure S2: Profiles of trypsin-activated toxins derived from *Bt* isolates with toxicity to ACP, Figure S3: Identification of toxins in IBL-00200 by peptide sequencing, Figure S4: Identification of toxins expressed by strain IBL-00200, Figure S5: Profiles of trypsin-activated *Bt* toxins Cry1Ab and Cry1Ba with toxicity to ACP.
