# Supplementary material for: Toxicity of Bacillus thuringiensis-Derived Pesticidal Proteins Cry1Ab and Cry1Ba against Asian Citrus Psyllid, Diaphorina citri (Hemiptera)
Source: Toxins (Basel). 2019 Mar 22;11(3):173. doi: 10.3390/toxins11030173 (PMC6468527; doi:10.3390/toxins11030173)
Supplement: Supplementary file 1 [file toxins-11-00173-s001.pdf]

Supplementary Materials: Toxicity of *Bacillus thuringiensis*-Derived Pesticidal Proteins Cry1Ab and Cry1Ba against Asian Citrus Psyllid, *Diaphorina citri* (Hemiptera)

Maria Teresa Fernandez-Luna, Pavan Kumar, David G. Hall, Ashaki D. Mitchell, Michael B. Blackburn and Bryony C. Bonning

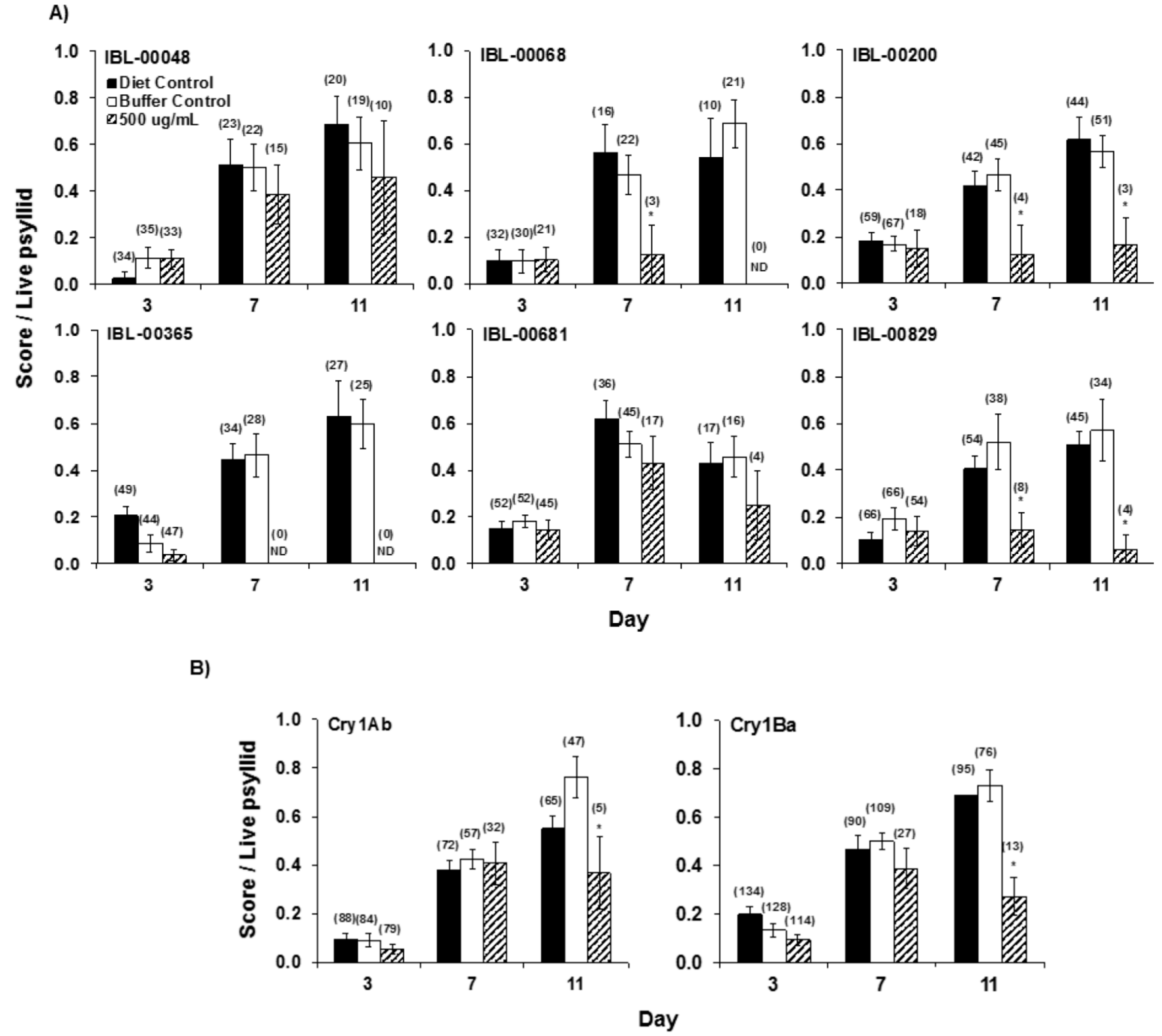

Figure S1. Toxicity correlated with reduced ACP excretions. Excretion score normalized to live animals at days 3, 7 and 11 of exposure to (**A**) *Bt* strains and (**B**) purified toxins at 500 μg protein/mL. Data are presented as Mean ± SEM. The number of psyllids scored is indicated (*n*). Significant differences between the test group and the Buffer control are indicated by * (Pairwise non-parametric Dunn's test, $p < 0.05$). ND: Not determined, due to 100% mortality.

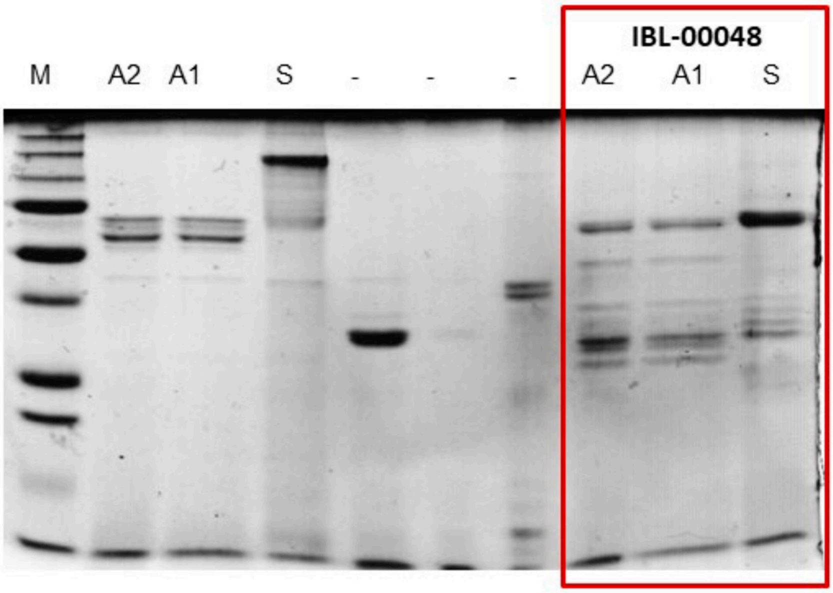
IBL-00048
M
A2
A1
S
-
-
-
A2
A1
S

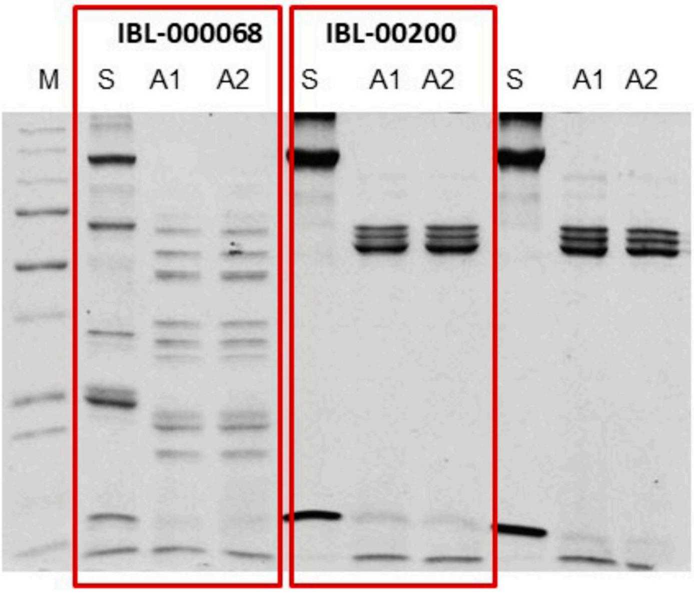
IBL-000068
IBL-00200
M
S
A1
A2
S
A1
A2
S
A1
A2

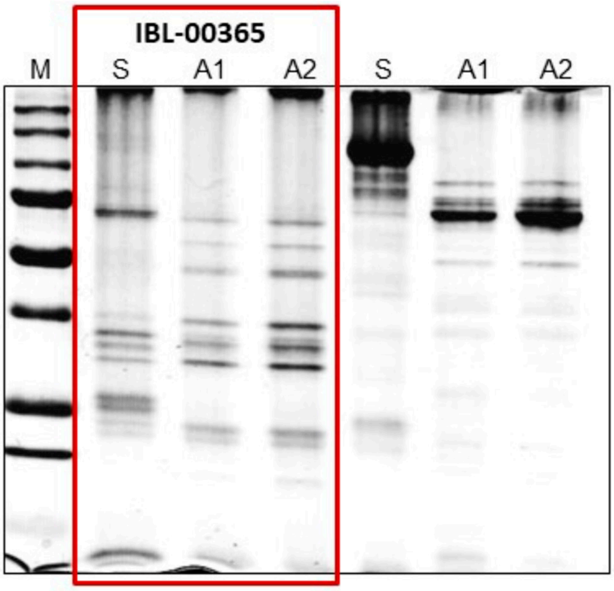
IBL-00365
M
S
A1
A2
S
A1
A2

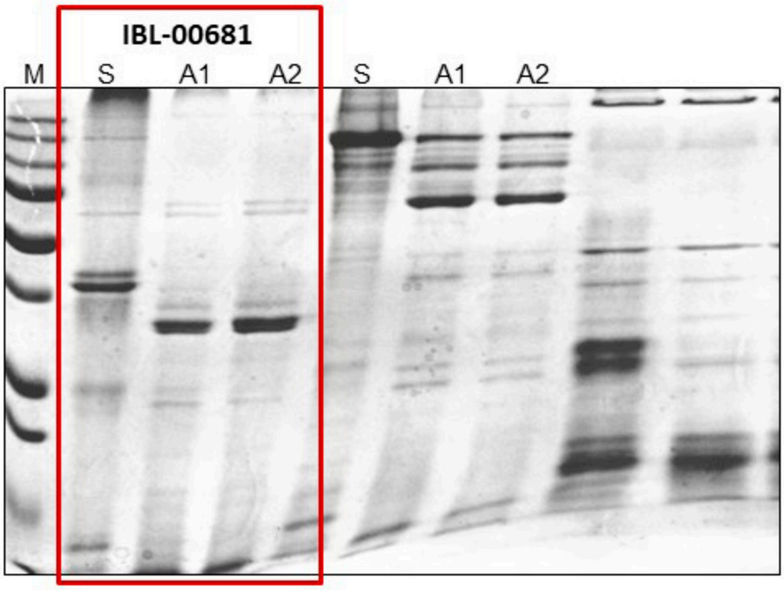
IBL-00681
M
S
A1
A2
S
A1
A2

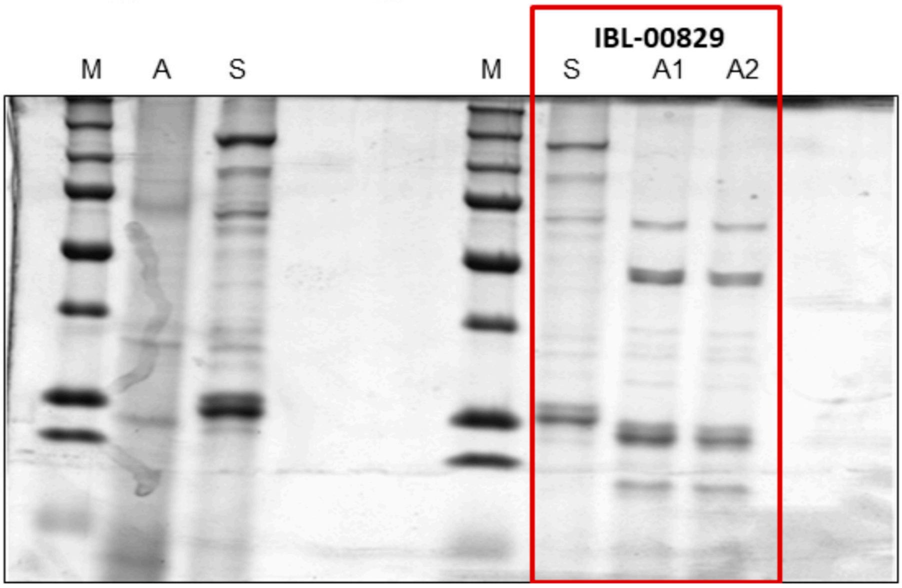
M
A
S
IBL-00829
M
S
A1
A2

Figure S2. Profiles of trypsin-activated toxins derived from *Bt* isolates with toxicity to ACP: Complete gels are shown for the cropped lanes in Figure 1. S: Soluble protein. A: Activated protein treated with 10% Trypsin for 1 h at 37°C. Proteins were separated by SDS-PAGE (12% gel) and stained with Coomassie Blue R. M, molecular mass markers.

Cry1Bb Band-A Coverage: 21.93 Score A7: 360.29
>gi|228848062|gb|EEM92927.1| Pesticidal crystal protein cry1Bc [*Bacillus thuringiensis* IBL 200]

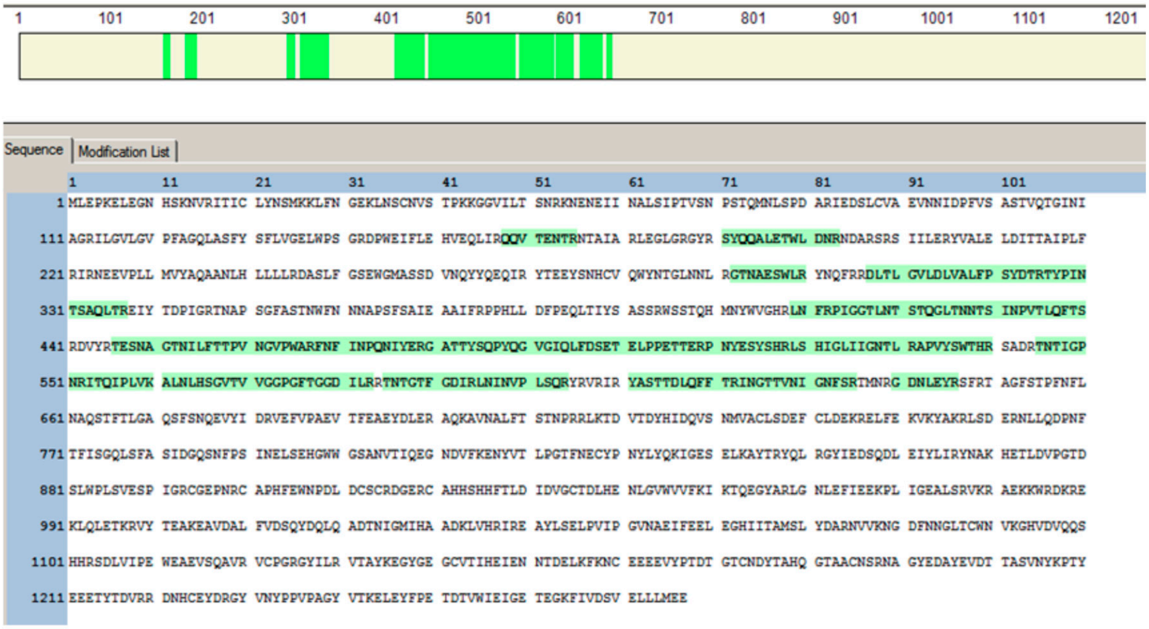

Cry1Bb Band-A Coverage: 21.93 Score A7: 360.29
>gi|228848062|gb|EEM92927.1| Pesticidal crystal protein cry1Bc [*Bacillus thuringiensis* IBL 200]

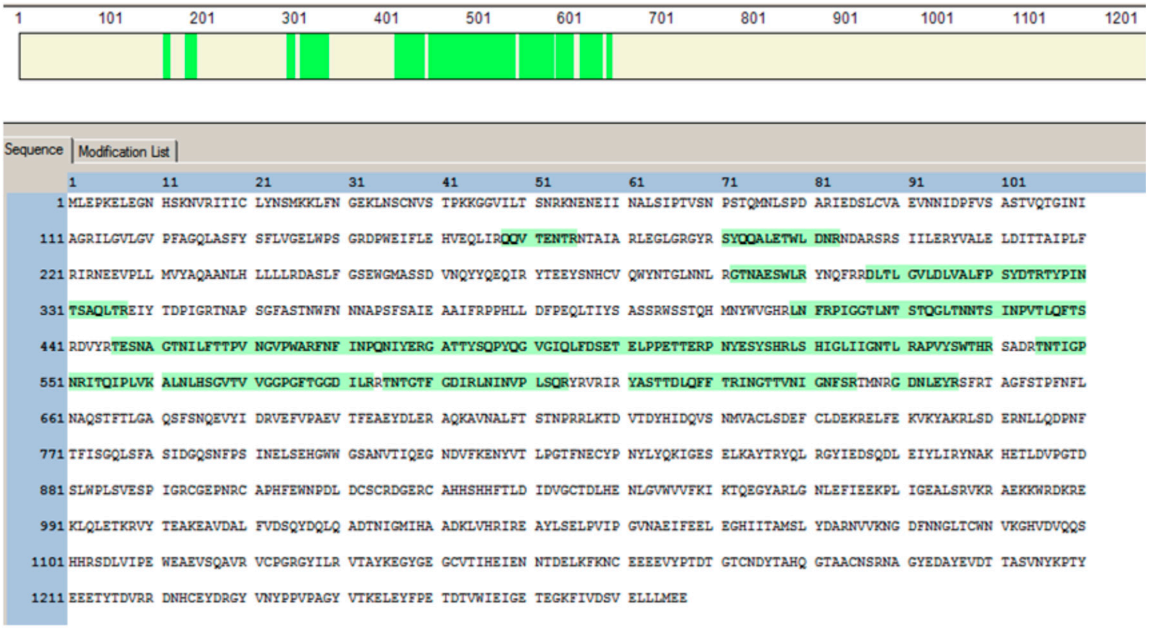

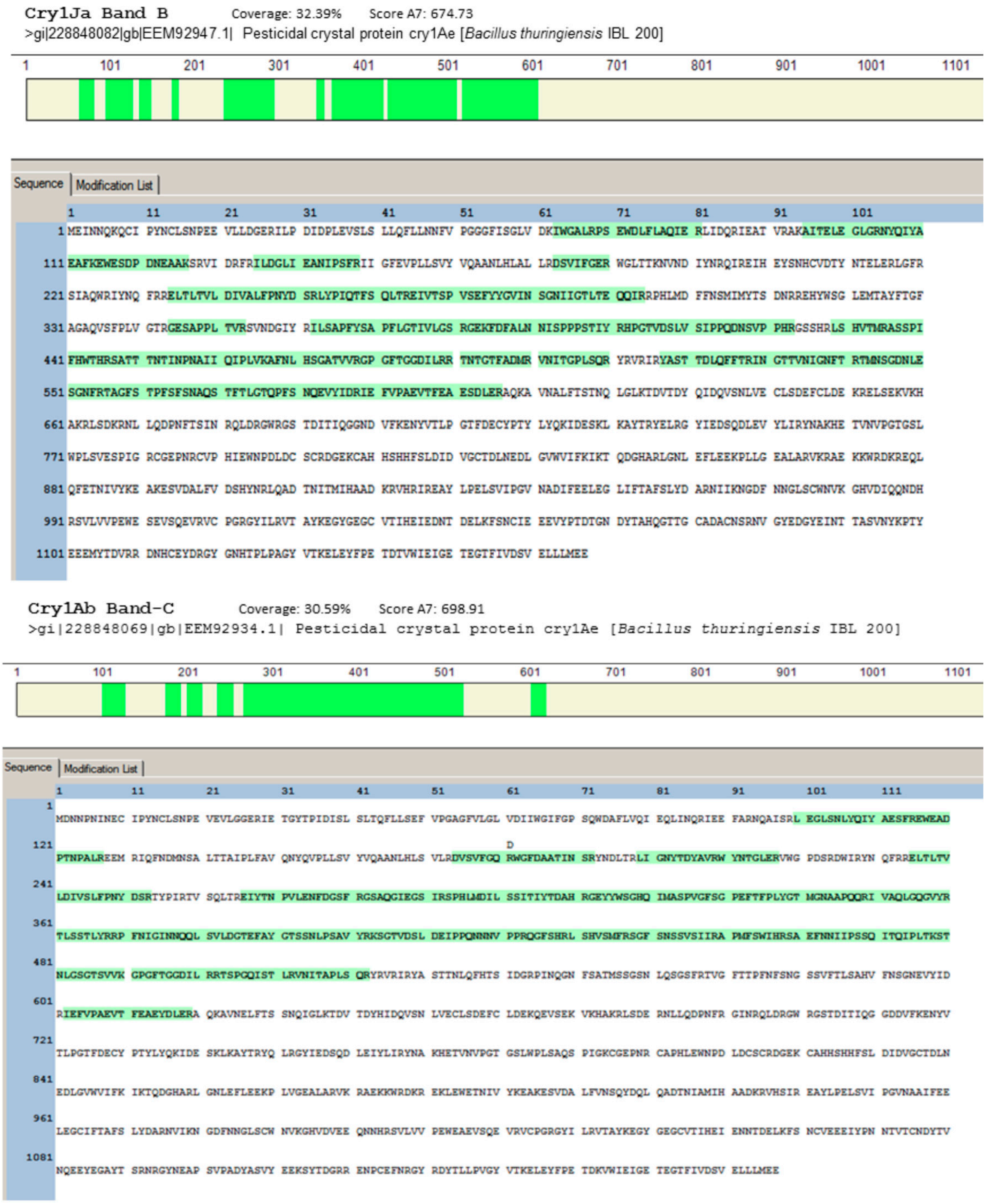

Figure S3. Identification of toxins in IBL-00200 by peptide sequencing. Peptide sequences obtained after trypsin treatment of bands A, B and C of strain IBL-00200 are highlighted.

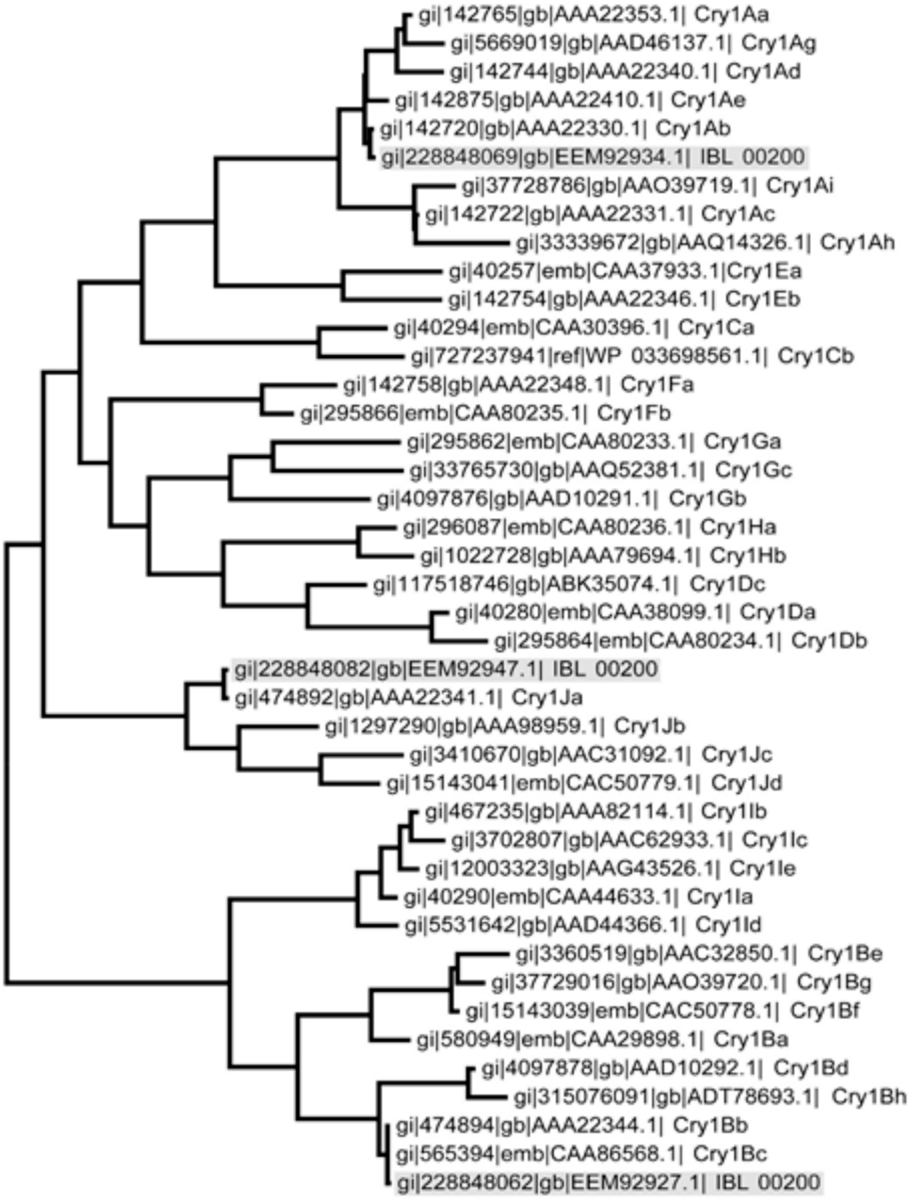

Figure S4. Identification of toxins expressed by strain IBL-00200. Two different Cry toxin sequences annotated as Cry1Ae cluster with Cry1Ab and Cry1Ja. One Cry toxin annotated as Cry1Bc clusters with Cry1Bb. Analysis performed with MEGA6 to generate a maximum likelihood tree.

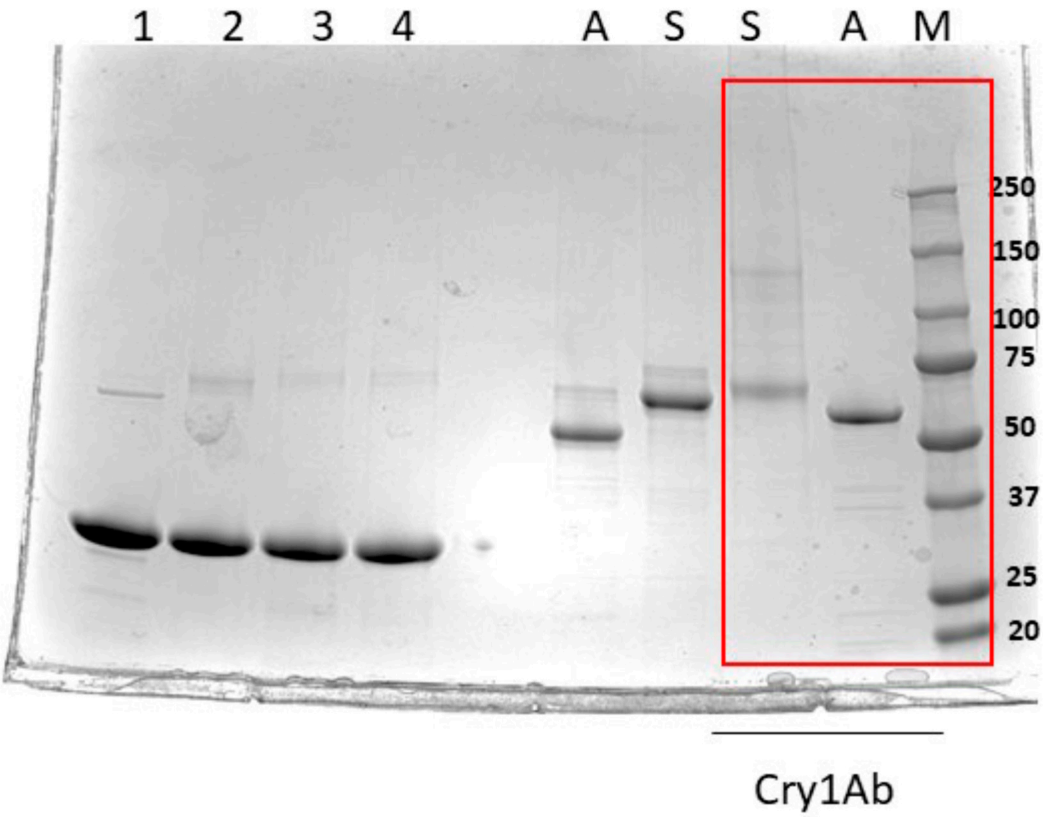

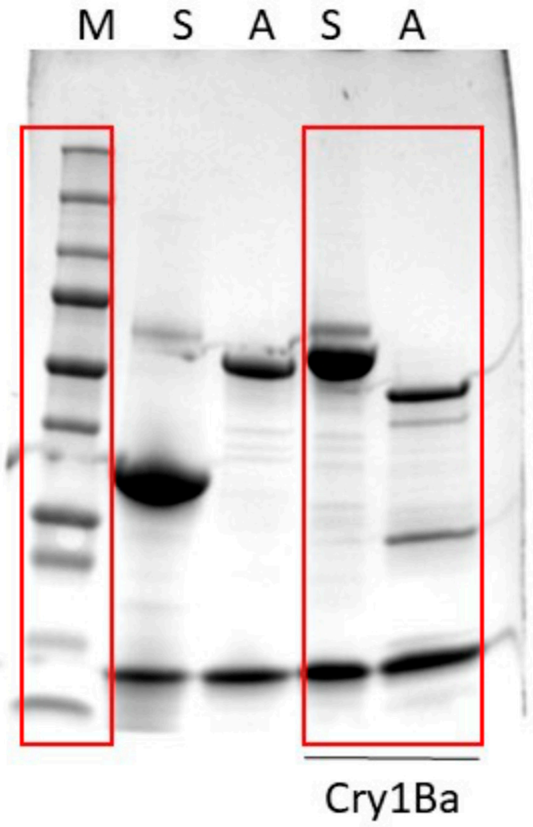

Figure S5. Profiles of trypsin-activated *Bt* toxins Cry1Ab and Cry1Ba with toxicity to ACP: Complete gels are shown for the cropped lanes in Figure 2. S: Soluble protein. A: Activated protein treated with 10% Trypsin for 1 h at 37°C. Proteins were separated by SDS-PAGE (12% gel) and stained with Coomassie Blue R. M, molecular mass markers.
